# Comparison of Efficacy and Safety of Low- Versus High-Dose Oral Prednisolone in Infantile Spasm (IS): An Open Label Randomized Controlled Trial at the Children’s Hospital & Institute of Child Health, Multan, Pakistan

**DOI:** 10.7759/cureus.23164

**Published:** 2022-03-14

**Authors:** Abdul Basit, Nuzhat Noreen, Syed Fawad Saleem, Muhammad Yousuf, Faisal Zafar

**Affiliations:** 1 Department of Pediatric Neurology, The Children’s Hospital and Institute of Child Health, Multan, PAK; 2 Department of Pediatric Neurlogy, The Children’s Hospital and Institute of Child Health, Multan, PAK

**Keywords:** hypsarrhythmia, weight gain, prednisolone, infantile spasm, hypoxic-ischemic encephalopathy

## Abstract

Background: Infantile spasm (IS) is an epileptic syndrome characterized by epileptic spasms, hypsarrhythmia on electroencephalography (EEG), and high risk of neurodevelopmental regression. This study was done to compare the efficacy and safety of the high versus the usual dose in children with IS.

Methodology: This open label randomized controlled trial was conducted at Department of Pediatric Neurology, The Children’s Hospital & Institute of Child Health, Multan, Pakistan, from January 1, 2020 to December 31, 2020. A total of 62 children (31 in each group) aged three months to two years presenting with epileptic spasms (at least one cluster per day) with EEG evidence of hypsarrhythmia were included. All 62 children were randomized to receive either high-dose prednisolone (10mg per dose four times a day) or the usual-dose prednisolone (2mg/kg/day thrice a day) for 14 days. Primary outcome measure was noted in terms of proportion of children who achieved complete, partial, or no response. Secondary outcome measure was proportion of children with adverse effects.

Results: In a total of 62 children, there were 34 (54.8%) male. Overall, mean age was noted to be 9.1±3.4 months. The most common etiology of IS was noted to be hypoxic-ischemic encephalopathy (HIE) in 28 children (45.2%). Significantly better clinical efficacy was reported in high-dose prednisolone group when compared to low-dose prednisolone cases as complete response, partial response and no response were noted in nine (29.0%), eight (25.8%), and 14 (45.2%) patients of low-dose group versus 18 (58.1%), eight (25.8%), and five (16.1%) patients in high-dose group, respectively (p=0.0265). Weight gain was the most frequently reported adverse effects noted in 11 (17.7%) cases. Overall, no statistically significant difference in the frequency of adverse effects (p=0.9573).

Conclusion: In comparison to low-dose prednisolone, high-dose prednisolone was found to be significantly more efficacious among cases of IS. Adverse effect in both treatment groups were relatively low and similar.

## Introduction

Infantile spasm (IS) is known to be an epileptic syndrome described by epileptic spasms, hypsarrhythmia on electroencephalography (EEG), and increased risk of neurodevelopmental regression. The IS is estimated to occur most commonly between age groups of three to 12 months and its overall incidence is between 0.3-0.4 per 1,000 live births while peak age of onset of IS noted to be between four and seven months [1,2]. Various causes of IS are labeled in the literature like structural, genetic, metabolic or perinatal causes [3,4]. Cessation of the spasms is the major aim of the treatment of IS whereas EEG is considered to an efficient tool to evaluate children with IS. Early treatment and cessation of spasm has been linked with improved neurodevelopmental outcomes among children IS [5]. On the other hand, delays in the treatment of IS have been associated with poor outcomes, including psychomotor regression and various other types of seizures in the later years of life [6,7].

Treatments for IS mainly include antiepileptic drugs (AEDs), corticotropic hormones, pyridoxine, and a ketogenic diet [8-10]. Conventional AEDs have been noted to result in unsatisfactory seizure control among children with IS [11,12]. Adrenocorticotropic hormone (ACTH) plays a major role in treating IS by suppressing endogenous corticotropin-releasing hormone (CRH) through a negative feedback pathway. ACTH is preferred as the first‐line treatment in most patients, but it is expensive and difficult to obtain [13,14]. As a result, the corticosteroids have been used effectively for decades [15,16].

No consensus exists regarding the best dosage regimen of prednisolone for the treatment of IS in children, but recently conducted research has shown that higher dosage of prednisolone might be more effective than conventional low dose of prednisolone (2mg/kg/day). Regional data showed that cessation spasm rates were better among children treated with higher dose of prednisolone versus the usual low dose (52% versus 25%, p=0.03), while side effects were relatively similar in both treatment groups [17]. In Pakistan, no randomized controlled trials have been done comparing low and high doses of prednisolone in children with IS. A higher dose of prednisolone might reflect higher rates of adverse effects as well, so we aimed this research to compare the efficacy and safety of the high versus the usual dose in children with IS.

## Materials and methods

Study Design

An open-label randomized controlled trial

Place and duration of the study

Department of Pediatric Neurology, The Children’s Hospital & Institute of Child Health, Multan, Pakistan from January 1, 2020 to December 31, 2020

Sample size

Considering two-sided confidence level (1-alpha) as 90%, power (% chance of detecting) as 80%, ratio of controls to cases as 1:1, proportion of controls with spasm cessation as 25%, and proportion of cases with spasm cessation as 51.6% [17], the sample size turned out to be 62 (31 in each group).

Inclusion criteria

Children with age between three and 24 months who presented with epileptic spasms with a frequency of a minimum 1 cluster/day with EEG confirmed hypsarrhythmia.

Exclusion criteria

Children with evidence of active tuberculosis, with active systemic illness, severe acute malnutrition as visible wasting/mid upper arm circumference < 11.5 cm [10], already taking steroids, having tuberculus sclerosis, or those with severe hypertension

Data collection

Approval from the Ethical Committee of the Children’s Hospital & Institute of Children Health Multan Pakistan was taken via letter number CHC/IEC/20-247. Informed and written consent was sought from all study participants. A total of 62 children as per inclusion and exclusion criteria were randomized to receive either high-dose prednisolone or the usual-dose prednisolone. Randomization was performed using lottery method. Children in the low-dose prednisolone group were given prednisolone as 2mg/kg/day thrice a day for 14 days. Children in the high-dose prednisolone group were given prednisolone as 10mg per dose four times a day, irrespective of weight, for 14 days. Contact numbers of parents/guardians of all enrolled children were noted, and they were reminded telephonically two to three days before of their 14-day follow-up date about the dates of their follow-up visits. Outcomes were noted at the end of 14-day treatment. A specialized proforma was made to record all study data. Demographic data including gender, age, weight, and area of residence were noted. Detailed history and physical examination was done. EEG findings were noted. Etiological diagnosis was recorded in all cases. At 14 days, primary and secondary outcomes were noted.

Outcome measures

Primary outcome measure was noted in terms of proportion of children who achieved spasm freedom for at least 48 hours (complete), more than 50% spasm reduction (partial), or less than 50% spasm reduction (no response) on Day 14. Secondary outcome measure was the proportion of children with adverse effects. Incidents of vomiting and irritability were asked of the parents/guardians/caregivers, while infections were labeled with the help of complete blood count examination, urinalysis, and chest x-ray. Serum electrolytes were used to observe electrolyte imbalance. Blood pressure was measured to note hypertension. Weight was measured by an electronic weighing machine.

Statistical analysis

Data analysis was performed using SPSS version 26.0. Qualitative variables were compared using chi-square test while quantitative variables were compared employing independent sample t-test. P value less than or equal to 0.05 was considered as statistically significant.

## Results

In a total of 62 children, 34 (54.8%) were male. Overall, the mean age was noted to be 9.1±3.4 months. There were 40 (64.5%) children belonging to rural areas of residence. The most common type of etiology of IS was noted to be hypoxic-ischemic encephalopathy (HIE) in 28 children (45.2%). Table 1 compares characteristics of children in both study groups. It was observed that there was no significant difference statistically (p>0.05).

**Table 1:** Characteristics of children in both study groups (n=62) SD: standard deviation, HIE: hypoxic-ischemic encephalopathy, CNS: Central Nervous System

Characteristics			Low-Dose Group (n=31)	High-Dose Group (n=31)	P-Value
Gender	Male		16 (51.6%)	18 (58.1%)	0.6098
	Female		15 (48.4%)	13 (41.9%)	
Age (months), Mean±SD			9.4±3.6	8.8±3.2	0.4906
Weight (kg), Mean±SD			7.2±2.4	6.8±2.8	0.5482
Systolic Blood Pressure (mmHg), Mean±SD			87±6	85±5	0.1591
Diastolic Blood Pressure, Mean±SD			58±6	57±5	0.4787
Area of Residence	Urban		10 (32.3%)	12 (38.7%)	0.5955
	Rural		21 (67.7%)	19 (61.3%)	
Age at the Onset of Spasm (months), Mean±SD			4.5±2.4	4.2±2.1	0.6024
Frequency of Types of Spasms		Flexor	24 (77.4%)	22 (71.0%)	0.9369
		Extensor	3 (9.7%)	4 (12.9%)	
		Mixed	3 (9.7%)	5 (16.1%)	
		Single	26 (8.9%)	25 (80.6%)	
		In Clusters	24 (77.4%)	22 (71.0%)	
Etiology		HIE	15 (48.4%)	13 (41.9%)	0.8445
		Brain Malformation	8 (25.8%)	8 (25.8%)	
		CNS Infections	6 (19.4%)	8 (25.8%)	
		Unknown	3 (9.7%)	2 (6.5%)	
Spasms/day, Mean±SD			12±8	10±7	0.2991
Fully Vaccinated			26 (83.9%)	28 (90.3%)	0.4486
Consanguinity Marriage of Parents			19 (61.3%)	22 (71.0%)	0.4208

Table 2 shows comparison of primary and secondary outcomes between high-dose and low-dose prednisolone cases at the end of the 14-day treatment period. Significantly better clinical efficacy was reported in high-dose prednisolone group when compared to low-dose prednisolone cases (p=0.0265). Weight gain was the most frequently reported adverse effect noted in 11 cases (17.7%). Overall, no significant statistical difference was reported in the frequency of adverse effects between both study groups as shown in Table 2 (p=0.9573). All infection cases were of mild severity and managed accordingly. No mortality was observed in this study.

**Table 2:** Comparison of clinical efficacy after 14 days of treatment in high-dose versus low-dose oral prednisolone EEG: electroencephalography

Outcomes		Low-Dose Group (n=31)	High-Dose Group (n=31)	P-Value
Clinical Efficacy (Primary Outcome)	Complete Response	9 (29.0%)	18 (58.1%)	0.0265
	Partial Response	8 (25.8%)	8 (25.8%)	
	No Response	14 (45.2%)	5 (16.1%)	
EEG Findings	Normal EEG with complete resolution of hypsarrhythmia	5/9 (55.6%)	10/18 (55.6%)	0.7097
	Resolution of hypsarrhythmia with persistence of background epileptiform discharge	3/9 (33.3%)	4/18 (22.2%)	
	Persistence of hypsarrhythmia	1/9 (11.1%)	4/18 (22.2%)	
Adverse Effects (Secondary Outcome)	Vomiting	2 (6.5%)	3 (9.7%)	0.9573
	Weight Gain	4 (12.9%)	7 (22.6%)	
	Hypertension	1 (3.2%)	3 (9.7%)	
	Irritability	4 (12.9%)	3 (9.7%)	
	Electrolyte Imbalance	2 (6.5%)	4 (12.9%)	
	Infection	4 (12.9%)	6 (19.4%)	

## Discussion

In the present study, baseline characteristics of both treatment groups were somewhat similar but overall time span between the first diagnosis and present treatment point was higher than what is usually reported in developed countries, which could be due to lack of health-seeking behavior in the local population. We also noted a male predominance among cases of IS, as 54.8% of the infants were male. This could be due to more attention given to male babies for health-related problems in our local population. Male predominance among cases of IS has been reported earlier by some researchers as well [13,18]. In this study, HIE was noted to be the most common type of etiology reported in 45.5% cases of IS, while brain malformations were observed in 25.8% cases. Large data from the United Kingdom Infantile Spasm Study (UKISS) revealed HIE, chromosomal, and cerebral malformations to be the most common types of etiologies behind IS reported in 10%, 8%, and 8% cases, respectively [3]. A regional study showed perinatal asphyxia to be the most common cause behind IS reported in 55.6% cases, which is similar to what we reported [17].

In the present study, overall efficacy in terms of complete response of prednisolone was observed in 43.5% of the total cases. Complete spasm freedom rates have been reported between 28% and 49% by different researchers with various dosage regimens of prednisolone [19,20]. We noted that infants in the high-dose group reported significantly better efficacy when compared to the low-dose group (complete response as 58.1% vs. 29.0%, p=0.0265). These findings are very similar to what was reported by Chellamuthu P et al, where the low-dose prednisolone group reported complete response in 25% cases in comparison to 51.6% in the high-dose prednisolone group (p=0.03) [17]. The literature reports rates of complete cessation of symptoms due to low-dose prednisolone as around 25% as well. The UKISS reported a high proportion of infants (70.0%) achieving spasm freedom [21]. Kossoff et al reported the high-dose oral prednisolone group as having complete spasm cessation in 67% infants, whereas EEG findings were also normal in 70% cases using high-dose prednisolone [22]. Hancock and Osborne found 71.4% infants having complete spasms freedom by the end of 14 days were on the high-dose prednisolone regimen [23]. Complete spasm freedom is reported in the present study among 58.1% cases using high-dose prednisolone, which is somewhat lower than what has been reported in earlier studies, but this could be because of relatively longer lag duration between onset of spasms symptoms and the actual time of presentation of these infants to our study center. Data from developed countries report an average duration of 25-45 days between onset of symptoms and initiation of treatment among cases of IS [24]. The prolonged lag time is an established factor that lowers response rates of IS treatment. Prolonged lag time is also a known prognostic factor for adverse neurodevelopmental outcomes [25].

Major concerns with the higher dosage regimen of prednisolone are the adverse effects. Weight gain has known to be an important adverse effect of prednisolone treatment. But, in this study, we noted that there was no significant statistical difference between low-dose and high-dose groups, which means that higher dose of prednisolone for the treatment of IS accompanies relatively similar rates of adverse effects, but as a higher dose of prednisolone has much higher rates of complete spasm freedom, this regimen can be preferred over the conventional low-dose approach. Our findings in terms of statistically similar adverse effects reported with high-dose prednisolone when compared to the low-dose regimen are consistent with those found by other researchers [21-23]. The intellectual prognosis among babies with IS is generally considered to be poor as many of these cases have neurological impairments before the spasms. Quick initiation of the treatment and care among cases of IS are advocated by most experts [26].

Limitations of the study

Absence of blinding and possible inaccurate reporting by the parents/guardians about the study data are some of the known limitations of this study. Sample size was also relatively small, so further studies involving large sets of IS cases from multiple centers can further verity the findings of the present study. As we only noted relatively short-term outcomes in our cases, further studies incorporating long-term follow-up plans and dose adjustments during the treatment period can further enlighten us. We were also not able to perform video EEG recording for the documentation of spasm frequency, as has been the case in many of the studies conducted in the developed countries.

## Conclusions

In comparison to low-dose prednisolone, high-dose prednisolone was found to be significantly more efficacious among cases of IS. Adverse effects in both treatment groups were relatively low and similar. Further multicentral trials can help us evaluate the optimal dose of oral prednisolone as initiating treatment and the right dosing strategy will improve related outcomes in IS.
